# Computational Modeling Reveals that a Combination of Chemotaxis and Differential Adhesion Leads to Robust Cell Sorting during Tissue Patterning

**DOI:** 10.1371/journal.pone.0109286

**Published:** 2014-10-10

**Authors:** Rui Zhen Tan, Keng-Hwee Chiam

**Affiliations:** 1 A*STAR Bioinformatics Institute, Singapore, Singapore; 2 Mechanobiology Institute, National University of Singapore, Singapore, Singapore; Centrum Wiskunde & Informatica (CWI) & Netherlands Institute for Systems Biology, Netherlands

## Abstract

Robust tissue patterning is crucial to many processes during development. The "French Flag" model of patterning, whereby naïve cells in a gradient of diffusible morphogen signal adopt different fates due to exposure to different amounts of morphogen concentration, has been the most widely proposed model for tissue patterning. However, recently, using time-lapse experiments, cell sorting has been found to be an alternative model for tissue patterning in the zebrafish neural tube. But it remains unclear what the sorting mechanism is. In this article, we used computational modeling to show that two mechanisms, chemotaxis and differential adhesion, are needed for robust cell sorting. We assessed the performance of each of the two mechanisms by quantifying the fraction of correct sorting, the fraction of stable clusters formed after correct sorting, the time needed to achieve correct sorting, and the size variations of the cells having different fates. We found that chemotaxis and differential adhesion confer different advantages to the sorting process. Chemotaxis leads to high fraction of correct sorting as individual cells will either migrate towards or away from the source depending on its cell type. However after the cells have sorted correctly, there is no interaction among cells of the same type to stabilize the sorted boundaries, leading to cell clusters that are unstable. On the other hand, differential adhesion results in low fraction of correct clusters that are more stable. In the absence of morphogen gradient noise, a combination of both chemotaxis and differential adhesion yields cell sorting that is both accurate and robust. However, in the presence of gradient noise, the simple combination of chemotaxis and differential adhesion is insufficient for cell sorting; instead, chemotaxis coupled with delayed differential adhesion is required to yield optimal sorting.

## Introduction

Patterning of tissues is an important process in the development of multi-cellular organisms, necessary for the generation and correct organization of diverse cell types from undifferentiated progenitor cells. Tissue patterning functions both at the level of organisms, for example in anterior-posterior and dorso-ventral patterning to set up the correct body plan [1], [2], and at the level of organs, for example in the mouse limb [3]. Patterning of tissues by instructive signaling gradients generates spatial domains of discrete cell fates. The classic "French Flag" model relates the different cell fates to an external morphogen [4]. In this model, naïve cells exposed to a gradient of diffusible signal will adopt different fates as they experience different concentration of the signal. The “French Flag” model is appealing due to its relative simplicity. However, two conditions have to be fulfilled for the model to work. Firstly, the patterning morphogen has to be sufficiently precise to produce distinct cell-fates at cell-type boundaries. Due to the inherent stochasticity in molecular processes like production and transport of morphogens, noise in the morphogen gradient is expected [5], [6]. A large number of strategies have been proposed to explain how robustness can be achieved in the presence of a noisy morphogen gradient. Most of these strategies suggest approaches for better shaping the morphogen gradient [7], [8], [9], [10] like self-enhanced morphogen degradation and facilitated transport. Others focus on better detection of the morphogen [11], [12], [13] such as integration of signals from multiple morphogens and local cell-to-cell signaling. The second condition is that the cells have to maintain stable positions relative to the morphogen source to receive a correct concentration of the signal over time. However, this is unlikely as cell positions will possibly change due to cell migration and division.

Interestingly, such cell movements that are supposedly detrimental to the “French Flag” model have recently been proposed to be essential for an alternative model of tissue patterning [14]. In this model, different cell fates are first specified randomly and independently of cell position to produce a "salt and pepper" mixture. Subsequently, the mixture of cell types sort to form clusters of discrete cell fates. This model of patterning has been observed in *Dictyostelium* where cells randomly differentiate into prestalk or prespore cells that intermingle and then sort to form discrete prestalk and prespore regions [15], [16]. This model for patterning has also been suggested in higher organisms such as the chick otic placode and primitive streak [17], [18], [19], zebrafish pancreas [19] and mouse anterior head process notochord [20].

It has been difficult to validate the cell sorting model in these organisms due to the difficulty in performing time-lapse experiments to follow the precursor cells over time. However, recently, using *in toto* imaging of the zebrafish neural tube, Xiong *et al.* were able to dynamically follow neural tube development [21]. Contrary to the "French Flag" model, Xiong *et al.* found that progenitors of different fates were initially spatially mixed. The progenitors then moved and rearranged themselves into sharply delineated domains. This result suggests a more general role for the cell sorting model beyond simple organisms.

Although the cell sorting model has been proposed based on experimental observations, it remains unclear what the sorting mechanism is. Two different mechanisms, chemotaxis and differential adhesion, have been proposed [21], [22]. The role for chemotaxis in branching morphogenesis is also well documented. An example is the PTEN modulated chemotaxis in morphogenesis of the developing kidney [23]. There are also evidences of cell adhesion molecule playing important roles in sorting. Examples include DdCaD-1, a *cadA* gene in *Dictyostelium*, whose deletion led to aberrant prestalk and prespore cell sorting [24], and protocadherin in zebra fish somite segmentation [25].

In the chemotaxis model, the cells will first adopt cell fates completely independent of position and hence morphogen concentration. These cells have different chemotactic response to the morphogen gradient based of their fates. Cells of certain fates will express certain proteins enabling them to migrate strongly towards the morphogen gradient, whereas cells of other fates will express other proteins which make them less strongly attracted or even be repelled by the gradient. Thus, cells that are most strongly attracted to the morphogen gradient will end up closest to the morphogen source, followed by the cells that are less strongly attracted and, finally, by cells that are repelled by the gradient, leading to cell sorting.

The other model is differential adhesion following imperfect cell fate specification to a noisy morphogen gradient [21], [22]. In this model, unlike in the chemotaxis model where cell fates are specified independent of position, cells will experience a morphogen gradient that biases the fates that they adopt. However, perfect patterning does not occur due to noise in the gradient and as a result, a fraction of the cells will be specified incorrectly. These misspecified cells express different levels or types of surface adhesion molecules from their correctly-specified neighbors. Given a sufficient number of correctly specified progenitors, differential adhesion could potentially lead to sorting of the misspecified cells. The potential role of differential adhesion in the zebrafish neural tube development is supported by the cadherin depletion experiment [21] where perturbing cadherin-2, cdh-2, using morpholino and a dominant-negative version of cdh2 leads to more mixing of cell types. Differential adhesion has also been found to mediate migration and pool sorting of postmitotic neurons [26], and to coordinate cell migration in the intestinal crypt [27].

In this article, we used computational modeling to study the roles of the two mechanisms, namely chemotaxis and differential adhesion, in cell sorting. In particular, we used the Cellular Potts Model, CPM, [28], [29], [30] which has been widely used to study the effects of chemotaxis and differential adhesion [31], [32], [33], tumor growth [34] and signaling [35]. Related to our work, Jiang *et al.* studied mound formation of *Dictyostelium discoideum* and suggested possible cooperation between differential adhesion and chemotaxis to bring about a tip containing only pre-stalk cells [31]. They found that differential adhesion aided in the sorting of pre-stalk cells to the surface of the mound whereas chemotaxis functioned in tip formation. Thus, differential adhesion alone can lead to cell sorting without chemotaxis. Käfer *et al.* studied cell sorting in the presence of both differential adhesion and chemotaxis [32]. Their work aimed to provide a general framework for understanding cell rearrangement not specific for a particular biological context. They considered homogeneous chemotactic response where two cell types responded similarly to the chemotactic gradient. Finally, Zhang *et al.* studied in detail how the choice of cell adhesion molecule binding affects cell sorting [33]. They looked at the kinetics of cell sorting in the context of gradual vs. sharp changes in the expression of cell-cell adhesion molecules. They also discussed the role of interfacial tension in determining the sharpness of the boundaries of the sorted clusters. Their study helped connect signaling models at the molecular level to cell sorting and tissue patterning at the tissue-level.

Our model builds on these previous models [31], [32], [33] by focusing on a specific biological system, which is that of tissue patterning to generate sharply delineated regions of cell types. We also considered the effects of noise in the morphogen gradient and whether robust cell sorting can still occur. We also developed and calculated from our model quantitative metrics such as the fraction of correct sorting, fraction of stable sorting and sorting time very carefully. These metrics relating to the stability of the clusters and the time spent in sorting allowed us to study the robustness of boundary maintenance and the developmental time frame, which are pertinent issues when studying pattern formation.To do this, first, we determined whether either of the mechanisms alone can achieve cell sorting. For each model, we simulated multiple runs for a range of chemotactic and differential adhesion parameters. From the simulations, we assessed the performance of each mechanism by quantifying the fraction of correct sorting, the fraction of stable clusters after correct sorting, time taken for correct sorting and the size variations of the cells having different fates. Next, we determined cell sorting performance in the presence of both chemotaxis and differential adhesion.

Our main finding is that chemotaxis and differential adhesion confer different advantages to the sorting process. By incorporating both chemotaxis and differential adhesion into the model, we found that optimal - robust and accurate - sorting requires a combination of both chemotaxis and differential adhesion. In the absence of morphogen gradient noise, a simple combination of the two is able to achieve optimal sorting, whereas in the presence of noise, chemotaxis coupled with delayed differential adhesion response is required.

## Materials and Methods

### Model description

We used a two-dimensional lattice model based on the Cellular Potts Model, CPM, to understand the role of chemotaxis and differential adhesion in cell sorting. CPM is based on energy minimization. At each step of the simulation, the new total energy obtained by making a certain change to the system is computed and compared with the previous total energy. If the step leads to a decrease in the total energy, it will be accepted. Otherwise, the step will be accepted based on a metric calculated from the increase in total energy. In general, the smaller the increase in total energy, the more likely for the step to be accepted.

In the model, each cell is assigned a unique cell identity: 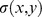, 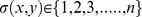 where 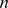 is the number of cells in the system and 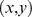 identifies a lattice site, 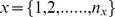 and 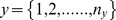, where 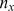 and 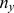 are the number of lattice points in the 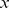
- and 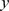
- directions respectively. Each cell is made up of several adjacent lattice sites that have the same cell identity. Cells in the model can belong to different types, 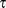, 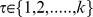, where 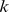 is the number of different cell types.

The energy of the interactions among cells in the CPM can be defined by the energy function



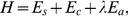
(1)


where 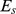 and 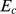 are the cell-type dependent surface energy and chemical energy, respectively, and 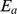 is an area-dependent energy term to maintain the area of the cells with 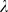 specifying the strength of the area constraint. We will now explain each of the terms in the energy function.

First, 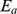 is given by
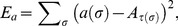
(2)


where 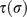 is the cell type associated with the cell 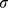, 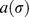 is the current area of a cell 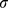 and 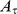 is the target area for cells of type 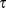. Having this area-dependent energy term allows the cells to be maintained within a fixed range of sizes.

Next, 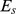 is given by
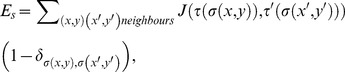
(3)


Here, 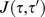 is the surface energy per unit contact area. It is defined as a function of the cell types (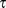 and 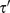) of the two surfaces in contact. The Kronecker delta term 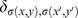 ensures that this energy is zero within a cell. To implement differential adhesion, 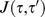 for 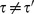 will be larger than 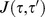 when 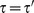. This will energetically favor configurations where cells of the same types are sorted together.

Finally, 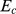 characterizes the energy in response to extracellular morphogen concentration, 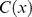, of the chemotactic signaling molecules. It is given by
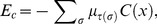
(4)


where 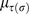 is the chemotactic potential of cell type 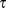.

At the start of each simulation, cell fates are first assigned. The fate assigned to a cell is based on a random number drawn for the cell and the cell's probability of adopting the various fates. In the chemotaxis model, cells are assumed to take on cell fates randomly, independent of position, and then sort depending on their different chemotactic response. Hence, in this model, each cell is assigned to have equal probability, 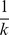, for taking on each of the possible 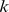
cell fates. On the other hand, in the differential adhesion model, cell fate specification is dependent on position but imprecise causing some cells to be specified incorrectly and have to be sorted out to yield correct sorting.

We divide the grid equally into 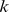 regions along the 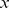-direction which is also the direction of the morphogen gradient. If cell fate specification is perfect, all the cells in the first region will adopt fate 1, and all the cells in the second region will adopt fate 2 and so on.

For imperfect cell fate specification, we define, *r*, an error ratio of specification, as
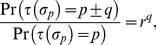
(5)


where 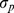 is a cell with its centroid in the *p*-th region and spanning the (*p−q*)-th to the (*p+q*)-th region, and 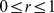. In this model, cells that are wrongly specified have the highest probability of being specified to take on the cell fates of adjacent regions. For 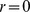, cell fate specification is perfect and for 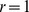, the cells adopt cell fates randomly. In the chemotaxis model, we use 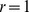. A small value of 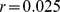 will be used for the differential adhesion model to introduce a small probability of error during cell fate specification.

After cell fate specification, the Metropolis Monte Carlo method is used to solve for the dynamics of the two-dimensional CPM model. At each step, a lattice site 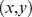 is chosen at random. Then, a neighboring site 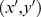 from its four neighbors is selected randomly. The value of 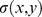 will be updated to the value of 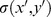 with the Monte Carlo probability, *p*,



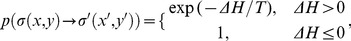
(6)


where 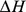 is the energy change for the substitution and 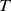 is the temperature that corresponds to the amplitude of cell membrane fluctuations. Time is measured in terms of Monte Carlo time step (MCS) with each time step defined to be the number of lattice points in the array.

### Model parameters

Our model consists of 100 cells on a 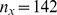 x 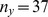 (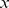
- and 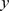
- directions) grid. The target area 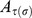 is set to be set to be 49 pixels, independent of cell type. Lattice points at the boundaries of the grids are inaccessible to the cells. These points are included for convenient calculation of 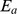. Reflecting boundary conditions are used. We set 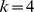 to have four types of cells. The constraint 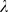 is set to 0.2 while 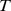 is set to 1.

The matrix 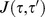 characterizes the surface energy per unit contact area among the four different types of cells. We set 
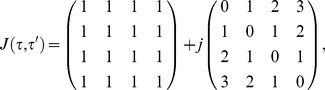
(7)


where 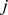 characterizes the strength of the differential adhesiveness among cells of different types. We assume that the differential adhesiveness of the cells types vary with their distances apart after correct sorting. For example, cells of type 2 are adjacent to cells of types 1 and 3 hence they exhibit the same magnitude of differential adhesiveness to both types of cells. On the other hand, cells of type 2 are further away from cell type 4, therefore the differential adhesiveness between the two types is larger.

The vector 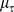 describes the chemotactic potential of the different cell types,
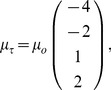
(8)


where 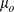 characterizes the strength of the chemotactic response. Here, cell types 3 and 4 are attracted to the gradient whereas cell types 1 and 2 are repelled by the gradient.

For the chemotaxis model, simulations are run for 100000 MCS whereas for the differential adhesion model, stimulations are run for 200000 MCS, due to the longer time taken for sorting in the differential adhesion model.

### Quantification of sorting performance

For each run, 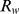, where 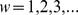 refers to each of the individual runs, we determine the number of clusters after every 100 MCS to obtain the number of clusters as a function of time, 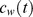. A cluster is defined as a connected group of cells of the same type. A run is considered to yield correct sorting if it is able to yield 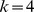 clusters at some point during the simulation.

This is given by



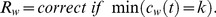
(9)


Hence the fraction of correct runs, *F_C_*, is given by 
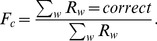
(10)


For each correct run, we determine the sorting time, 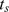, by finding the minimum time where 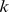 clusters are observed,
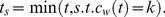
(11)


A correct run is also considered stable if it maintains the 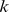 cluster throughout the simulation till the end, 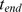.



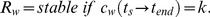
(12)


We define the fraction of stable runs, 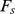, as
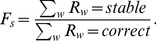
(13)


The cell size variation among the cells of different fates is characterized by first determining the mean size of cells with each cell type, 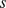, 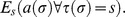


Cell size variation is then defined as the ratio
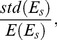
(14)


where *std* and *E* are the standard deviation and mean respectively.

### Quantification of how initial number of clusters affects sorting outcome

We examine how the initial number of clusters affects the sorting outcome and sorting time for simulations incorporating both chemotaxis and differential adhesion with a completely random initial assignment of cell fates. We examine the results separately for the various values of 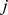 and 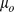. For each set of 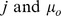 and 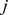, we first divide the runs into two groups, namely those that lead to correct sorting and those that lead to incorrect sorting. For certain values of 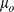 and 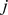, all the runs lead to either correct or incorrect sorting and hence do not have values for both groups hence sorting outcome analysis cannot be perform for these sets of 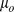 and 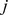.

To determine if the initial number of clusters for the two groups are significantly different, we perform a 2-sample t-test for values of 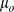 and 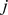 where there are at least two runs in each group. To determine whether there is a correlation between the initial cluster number and sorting time, we perform a spearman correlation test for values of 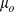 and 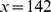 with at least three runs leading to correct sorting.

### Morphogen generation

We consider the simplest model for morphogen gradient generation, consisting of signaling molecule synthesis at one end of the cell grid, 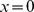, and diffusion away from the source towards 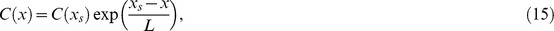. In addition, there is spatially uniform degradation by a first-order reaction. The steady-state solution to this model is an exponential decay, 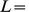
(15)


with 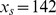, being a characteristic decay length of the diffusion, 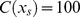 being the location of the source, and 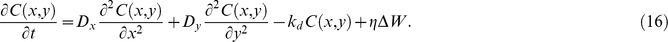.

### Noisy morphogen generation

In the last section of the paper, noise is introduced into the morphogen gradient to explore the sorting process in the presence of noise.

To introduce noise into the morphogen gradient, we add an extra term into the reaction-diffusion equation governing morphogen concentration,
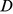
(16)


Here, 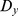 and 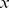 are the diffusion coefficients in the 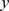 and 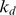 direction respectively, 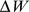 is the rate of degradation of the morphogen, 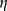 is Gaussian noise of mean 0 and variance 1, and 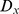 characterizes the magnitude of noise in the system. We assume that 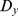 and 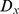 are equal, and choose 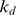 and 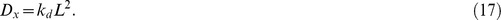 such that
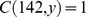
(17)


To solve equation (16), we use the implicit Euler scheme and set the following boundary conditions:
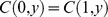


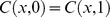


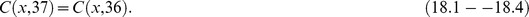


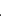
(18.1--18.4)


For each run, we first let the morphogen gradient evolved from the deterministic noiseless gradient for 50000 steps before starting the cell sorting process. After that, we update the noisy gradient at every MCS.

### Quantification of sorting time for temporal control of differential adhesion

For runs with temporal control of differential adhesion, 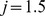 for the first 500 MCS and 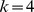 in the subsequent 1500 MCS. In this case, a run is considered to yield correct sorting if it is able to yield 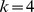 clusters after the first 500 MCS during the simulation. This is to take into account that even if a run is able to yield 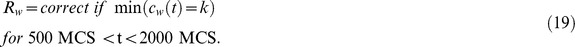 clusters during the first 500 MCS, the 4 clusters will be unstable and likely to dissociate in the presence of noise.

Hence for these runs,



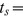
(19)


The sorting time, *t_s_*, is


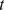
 for 500 MCS <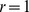 <2000 MCS. (20)

## Results

### Chemotaxis leads to correct but unstable sorting

**Figure 1:**
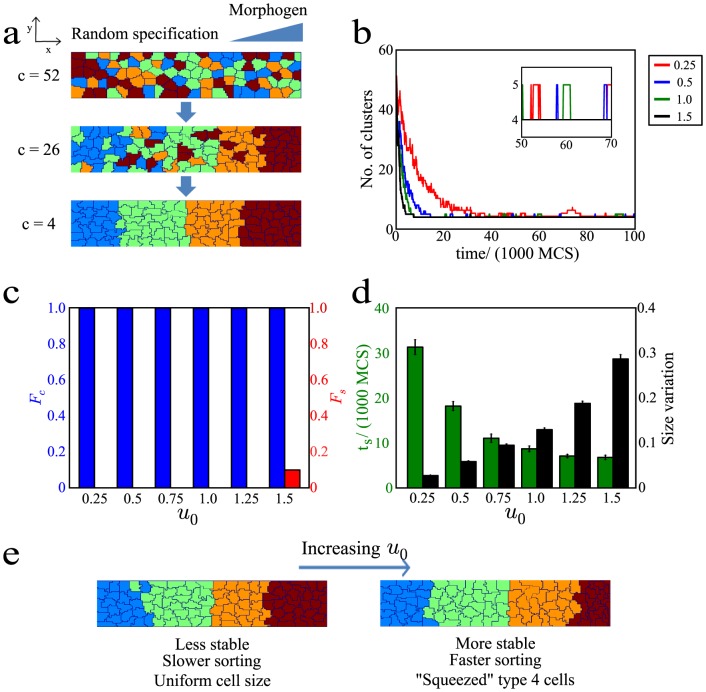
Chemotaxis model leads to high fraction of correct sorting but low level of stable sorting. 10 runs were performed for each value of *μ_o_*. (**a**) The cell grid at start (top), middle (middle) and end of a run (last). The number of clusters, 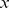, is shown to the left of the grid. Cell boundaries are in dark blue. The morphogen source is located along the rightmost lattice points (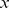  =  142) and diffuses to form a gradient in the 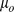-th (horizontal) direction. The different cell types are colored in light blue (type 1), green (type 2), orange (type 3) and red (type 4). (**b**) Plot of number of clusters with time for different values of magnitude of chemotactic strength, 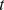. The number of clusters decreases steadily with time. (Inset) Zoom-in plot of number of clusters between 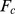  =  50,000 and 70,000 MCS. (**c**) Bar graphs of fraction of correct and stable sorting, 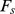 (blue) and 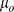 (red), respectively, for different values of 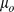. For 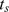 <1.5, none of the runs led to stable sorting. (**d**) Bar graphs for sorting time, 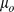, (green) and size variation (black) for different values of 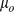. Error bars show the standard errors. (**e**) Summary of findings for low and high 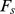.

First, we explored the dynamics of chemotaxis and determined if this mechanism can lead to correct sorting of the cells into four regions of differing magnitude of the chemotactic response. We set 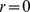, corresponding to a completely random initial assignment of cell fates (Fig. 1a, top). (Recall that 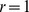 corresponds to perfect cell fate specification while 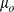 corresponds to the cells adopt cell fates randomly.) After this assignment, each cell would respond to a deterministic gradient with the chemotactic response depending on its fate. To examine how the sorting progresses with time, we quantified the number of clusters at each time point. At the start of the stimulation, the number of clusters was high, owing to the random assignment of cell fates. As time progressed, the cells sorted out according to their differences in chemotactic potential. This led to a steady decrease in the number of clusters. Correct sorting was eventually achieved when the number of clusters obtained is equal to the number of cell types (Fig. 1a). To study the sorting response to different chemotactic potential, we varied the magnitude of chemotactic strength, 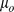, and plotted the number of clusters for different values of 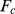 (Fig. 1b). We found that although the fraction of correct sorting, 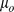, is 1, independent of 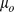 as shown in Fig. 1c, increasing 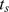 led to a decrease in the sorting time, 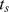, as shown in Fig. 1b and 1d. Furthermore, the standard deviation for 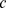 from different independent runs is small. This demonstrates that the chemotaxis model is a reliable mechanism for achieving correct sorting.

However, when we examined the temporal trace of the number of clusters more closely, as in the inset of Fig. 1b, we observed that the correct clusters obtained were not stable as spontaneous dissociation of the correct cluster occurred intermittently. This usually happened when one cell at the edge of a cluster dissociated from the cluster and mixed with cells from another cluster for a brief period of time. This is detrimental to development as clear boundaries among the different types of cells will not be properly maintained. To characterize the stability of the sorting, we quantified the fraction of stable runs, 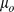, as the fraction of correct runs that maintains the correct number of clusters throughout the simulation. As observed in Fig. 1c, none of the runs for 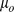 <1.5 are stable and about 20% of the runs for 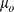 =  1.5 are stable. This showed that, with larger values of 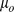, the four clusters can be stably maintained. We quantified the lifetime of the correct clusters between their intermittent breakup (Fig. S1 in File S1) and found that the lifetime of the clusters increases with 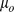. This was consistent with the higher fraction of stable runs at higher values of 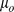 However we found that the size variations among the four types of cells increased with 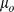 (Fig. 1d). This is largely due to the fact that at high values of 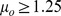, 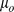, the effect of the gradient on cells of types 3 and 4 tend to be stronger, causing them to become "squeezed" as they approached the morphogen source (Fig. 1e). This is similar to the size-based segregation of cells observed in other cell sorting simulations [32].

In summary, low magnitude of the chemotactic response 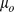 leads to slower sorting, more uniform size of the different cell types and higher spontaneous dissociation, whereas high 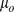 leads to faster sorting, greater variation in cell sizes and lower spontaneous dissociation (Fig. 1e). Hence an intermediate level of 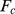 can balance the trade-off between size variation and spontaneous dissociation.

In the above chemotaxis model, some cells were repelled whereas others were attracted to the morphogen. We repeated the analysis to determine if sorting can occur if the different types of cells responded in the same way to the morphogen but with different magnitudes for both attractive and repulsive responses. As shown in Fig. S2a and S2c in File S1, purely attractive or repulsive responses can lead to a high fraction of correct sorting, 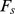. Similar to the results before, the fraction of stable runs, 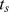, is low. Furthermore, the responses of sorting time, 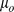, and size variation to increasing 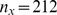 remain unchanged. (Fig. S2b,d in File S1).

To rule out boundary effects and test for the generality of our conclusions, we repeated the analysis on a larger grid, 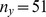 x 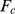, with 210 cells, as compared to the current grid with 100 cells, keeping all the other variables constant. Consistent with the current model with fewer cells, high fraction of correct sorting, 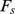 was attained whereas fraction of stable runs, 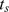, was low. (Fig. S3a in File S1) As expected, the sorting time, 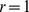, increased with the increased number of cells. Furthermore, the size variation also increased as the cells in the larger grid experienced a larger range of the chemotactic gradient as the characteristic decay length of diffusion and the concentration of the morphogen at the source were kept constant (Fig. S3b in File S1). Since the results from grids of different sizes are qualitatively similar, we conclude that our results are independent of cell number.

### Differential adhesion leads to low occurrence of correct and stable sorting

**Figure 2:**
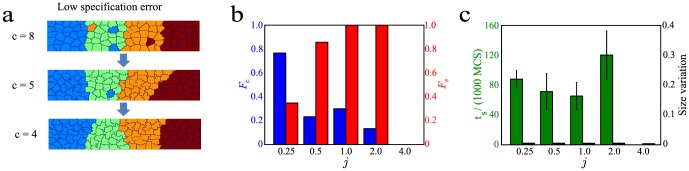
Differential adhesion model leads to low fraction of correct sorting but high fraction of stable sorting. 30 runs were performed for each value of 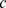. (**a**) The cell grid at start (top), middle (middle) and end of a run (last). The number of clusters, 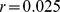, is shown to the left of the grid. The parameter 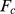 is used to mimic a low cell fate specification rate. (**b**) Bar graphs of fraction of correct and stable sorting, 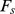 (blue) and 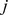 (red), respectively, for different values of magnitude of differential adhesion, 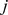. For 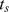  =  4, none of the runs lead to correct sorting. (**c**) Bar graphs for sorting time, 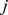, (green) and size variation (black) for different values of 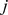. Error bars show the standard errors.

Next, we studied the dynamics of differential adhesion and determined if this mechanism can sort misspecified cells into their correct compartments. In this model, a noisy morphogen gradient patterns the cells to take on different cell fates. Due to noise in the gradient, a fraction of the cells will not be specified correctly. Hence, unlike the chemotaxis model with 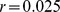 leading to a random initial assignment of cell fates, we set a low value of 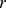. This low value of r allows most of the cells to adopt the correct fates and a small number of cells to be misspecified (Fig. 2a, top). Since there are 100 cells, this value of 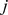 leads to approximately 2–3 misspecified cells.

We examined the sorting behavior for different values of the magnitude of differential adhesion, 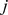, and quantified the fraction of runs where misspecified cells were sorted correctly. The initial number of clusters is low as most of cells are specified correctly (Fig. 2a). We simulated 30 runs for different values value of 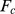
and quantified the fractions of correct and stable runs, 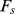 and 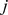, respectively. Many runs did not lead to correct sorting. Indeed, we found that differential adhesion is unable to achieve 100% correct sorting for the range of 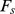 investigated (Fig. 2b). But interestingly, 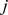 is much higher than that for the chemotaxis model, reaching 1 for high values of 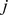. For 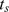 =  4, none of the runs led to correct sorting. Thus, runs that are correctly sorted, though rare, are almost always stable.

We quantified the sorting time, 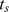, and found no clear relationship between 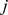 and 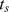 (Fig. 2c). Furthermore, 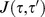 is much longer (∼ 60000 MCS as compared to ∼ 10000 MCS) and has a much larger variation than that of the chemotaxis model. These results suggested that, unlike chemotaxis, sorting by differential adhesion is slower and more stochastic. We also characterized the cell size variation among the different cell types and observed low variations (Fig. 2c). This is consistent with the absence of any force biasing the sizes of the different cell types.

In the above model, differential adhesion of the cells types vary with their distances apart after correct sorting as captured by equation (7). This is more stringent than a model whereby cells “dislike” cells of all other types equally. To test this model, we used a different matrix 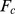 as shown in Fig. S4a in File S1. We compared 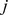 for the two different models for 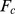 =  0.25, 1.0 and 4.0 and observed that the stringent model achieved higher fraction of correct sorting as compared to the second model (Fig. S4b in File S1). We examined the runs from the second model and realized that if a cell cannot discriminate among unlike cells, it is likely to be sorted to the boundaries between two different cell types as this is a local minimum in the energy function (Fig. S4c in File S1). Hence a simple model where cells dislike cells of all other types equally leads to poorer sorting.

### In differential adhesion model, cell speed affects correct sorting

**Figure 3:**
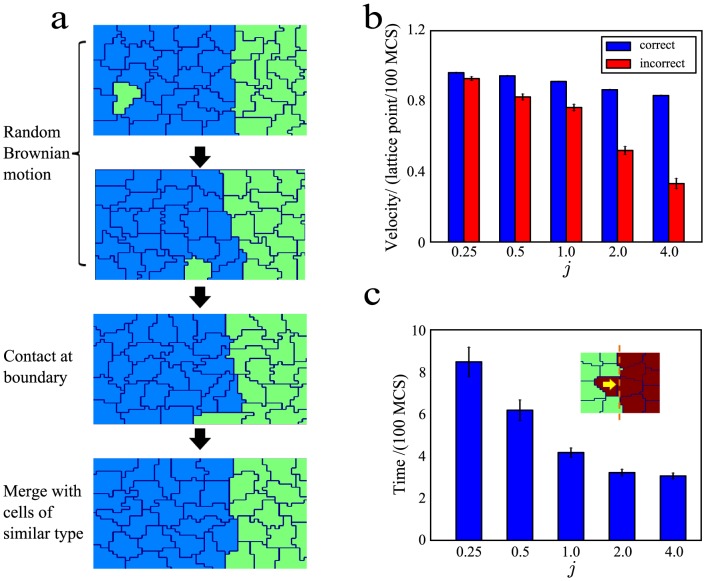
Speed of misspecified cells decreases with magnitude of differential adhesion, 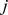
 **.** (**a**) The cell grid as time progresses (top to bottom). (**b**) Bar graphs showing the speeds of correctly specified (blue) and incorrectly specified cells (red) for different values of *j*. (**c**) Bar graph of the merging time for different values of 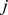. (Inset) Cell grid showing the initial configuration of the cells. The average time taken for the centroid of the misspecified to cross the boundary (dotted line in orange) is measured for 100 runs. Error bars show the standard errors.

As shown in Fig. 2b, the fraction of correct runs, 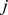, decreases with the magnitude of differential adhesion, 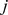. This result was unexpected as, intuitively, one would think that increasing the magnitude of differential adhesion among the different types of cells would lead to better sorting. To make sense of this unexpected result, we examined the sorting process more closely. During the sorting process, a misspecified cell will move among the cells that are specified correctly (Fig. 3a, top). This movement is random with no preferred direction as the misspecified cell is homogenously surrounded by correctly-specified cells. During this random movement, the misspecified cell may reach the boundaries of different clusters, thus coming into contact with cells of its own type (Fig. 3a, second from bottom) and moving into the cluster consisting of cells of its type (Fig. 3a, bottom).

Since the process of sorting involves the random motion of the misspecifed cell, the speeds of the misspecified cell will be an important determinant of whether correct sorting occurs. As the speed of the misspecified cell decreases, it has a lower probability of reaching the boundary. We estimated the speeds of correctly-specified and misspecified cells by calculating the absolute difference between their centriods at every 100 MCS. We found that the speed of correctly-specified cells is always higher than that of misspecified cells (Fig. 3b). This is expected as cells of the same type will intermingle more readily. Unlike the speed of correctly-specified cells which remained relatively constant, the speed of misspecified cells rapidly decreased with 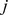 (Fig. 3b). This showed that the speed of cells decreased as its differential adhesion strength, 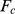, with its neighbor increased, leading to a lower fraction of correct sorting, 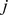. This was expected as the higher the value of 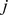 was, the more energetically costly it would be to deform the cell. This would lead to a much lower acceptance rate for simulations that change the cell edge at the boundaries of the misspecifed cells. This directly contribute to reduced effective movement of the misspecifed cells, causing their speeds to decreased with 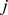.

The random motion of a cell among cells of another type is different from the situation when a cell is in contact with cells of both the same and different types. In the latter case, we will expect a higher value of 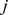 to facilitate the integration of the cell into the region of cells having the same fate. To test this hypothesis, we performed another set of stimulations with a smaller number of cells. In this simulation, there were only 16 cells, arranged in a 4 by 4 manner. 7 of the cells are of type 1 and the remaining 9 are of type 2. With this initial setup, we determined the average time in 100 runs needed for the cell marked with the arrow (Fig. 3c, inset) to cross the boundary highlighted by the orange dotted line for different values of 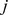. As expected, this time decreases with 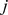 (Fig. 3c).This shows that when a cell is in contact with cells of the same and different types, having a larger 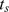 increases its adhesion with cells of the same type and speeds up its integration with cells of the same type. This integration time, of the order of 500 MCS, is much shorter than 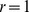, of the order of 80,000 MCS (Fig. 2d).

### Intermediate levels of chemotactic and differential adhesion responses yield high fraction of correct and stable sorting with low cell size variations

We have observed two different sorting outcomes based on the chemotaxis and differential adhesion models. We found that chemotaxis is better for achieving correct clustering whereas differential adhesion is better for stabilization of the correct clusters. Their different advantages suggest that some optimal combination of the two mechanisms may exist to attain both correct and stable sorting.

**Figure 4:**
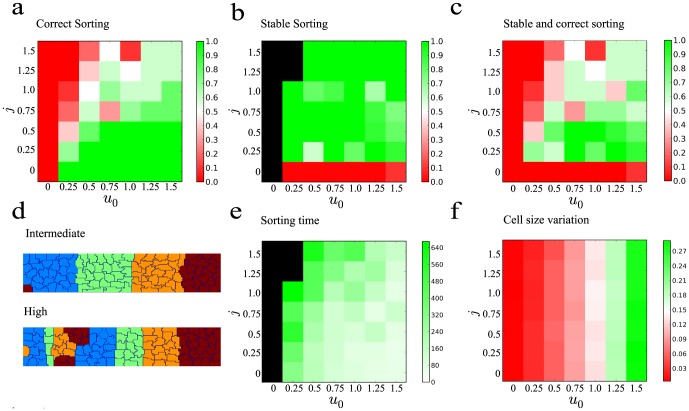
Intermediate levels of magnitudes of chemotactic response, 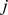, and differential adhesion, 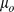, lead to correct and stable sorting with low cell size variations. 10 runs were performed for each value of 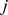 and 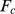. (**a**–**c**) Fraction of correct sorting, 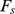 (a), stable sorting, 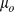 (b) and fraction of runs that lead to both correct and stable sorting (c) for different combinations of 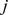 and 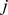. (**d**) Cell grid obtained for intermediate (top) and high 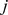
(last). For intermediate 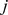, a few cells are sorted incorrectly. At high 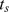, a larger number of cells are mis-sorted. These mis-sorted cells formed stable clusters with cells of the same fate. (**e**) Sorting time, 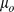, for different combinations of 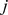 and 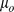. (Combinations of 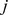 and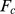 with 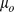 =  0 are shown in black) (**b**) Cell size variation for different combinations of 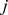 and 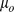. Size variation increases with 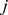 but does not depend on 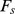.

To test this hypothesis, we determined the sorting response when the cells exhibit both chemotaxis and differential adhesion. Cells were initialized with 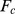, corresponding to a completely random initial assignment of cell fates. We plotted the fraction of correct sorting, 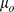, for different values of magnitude of chemotactic response, 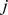, and magnitude of differential adhesion, 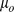 (Fig. 4a). We found that chemotaxis alone leads to a high fraction of correct sorting similar to results shown in Fig. 1c. On the other hand, differential adhesion alone is unable to lead to correct sorting. This is expected as the stimulation is started from a random configuration. Interestingly, for a fixed value of 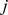, increasing the value of 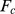 generally leads to a decrease in the fraction of correct sorting, 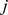 (Fig. 4a). This suggests that there is a "tug-of-war" between the chemotactic force of individual cells and the collective adhesion exerted by cells of the same type. To study this effect, we examined the cell arrangement for runs that did not lead to correct sorting (Fig. 4d). Most of the sorting errors occurred at the edge furthest away from the morphogen gradient where the gradient is most gradual. At intermediate values of 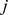, we often observed one or two mis-sorted cells stuck at the edge (Fig. 4d, top). This happened as the gradual chemotactic gradient at this edge was insufficient for the cell to overcome the stronger repelling force exerted by neighboring cells due to differential adhesion. At high values of 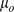, we observed higher number of cells being mis-sorted (Fig. 4d, bottom). These mis-sorted cells formed stable clusters with other mis-sorted cells of the same type. In this case, the adhesion of a cluster of cells of the same type impedes migration of the individual cells towards the morphogen source.

Next, we examined the fraction of stable sorting, 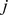. Certain combinations of 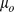 and 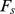 are unable to lead to correct sorting and hence, 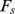 cannot be computed for these values. These combinations are shown in black in Fig. 4b. For the remaining combinations where correct sorting was achieved, we computed 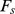. As shown in Fig. 4b, high 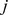 is achieved when the value of 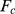 is non-zero, showing that introducing a small amount of differential adhesion can improve the stability of the clusters significantly.

Next, we determined the fraction of correct and stable runs by computing the product of 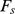 and 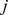. As shown in Fig. 4c, the highest fraction of correct and stable runs is observed for intermediate values of 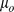 and intermediate and high values of 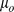. This is the regime whereby differential adhesion is low enough not to impede the migration of individual cells during the sorting process but is sufficient in combination with intermediate values of 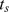 to maintain stability of the clusters after correct sorting has occurred.

We determined the sorting time, 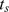, and the size variations of the cells for these runs with both chemotaxis and differential adhesion. We found that 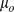 decreased with 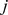
and increased with 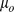 (Fig. 4e). This agrees with the observations above that higher chemotactic response leads to higher migration speed of individual cells whereas differential adhesion leads to adhesion of cells of similar cell fate thus impeding migration of individual cells. We found that size variation is a function solely of 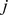 and does not depend on 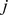 (Fig. 4f). We concluded that intermediate values of 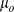 and 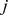 are optimal for achieving high fraction of correct and stable sorting with low cell size variations.

As shown in Fig. 4d and discussed above, mis-sorted cells could form stable clusters with other mis-sorted cells of the same type, thus impeding migration towards the morphogen source. This observation suggests that the initial arrangement of cells may play an important role in affecting the sorting outcome. Hence, we determined if the initial number of clusters would affect the sorting outcome and sorting time for the various values of 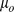 and 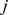. Since each simulation was initialized independently, the initial number of clusters could take on different values. Indeed, we observed that the initial cluster number ranged from 37 to 60 in our simulations. For our system of 100 cells, this would correspond to cell clusters of about one to three cells. To determine if initial cluster size could lead to differences in sorting outcomes, we performed a 2-sample t-test to determine if initial number of clusters for runs that led to correct sorting and those that led to incorrect sorting are significantly different for each values of 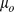 and 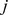. From the p-values shown in Table S1a in File S1, we concluded that no significant differences in initial cluster numbers were observed between runs that led to correct sorting and those that did not. Next, we determined whether there was a correlation between initials cluster number and sorting time by performing a spearman correlation test between the two variables. From the p-values shown in Table S1b in File S1, we concluded that no significant correlation was observed. The lack of influence of initial number of clusters on the sorting outcome is likely due to the small size of the clusters.

### Chemotactic response combined with later activation of differential adhesion response yield high fraction of correct and stable sorting in presence of morphogen noise

**Figure 5:**
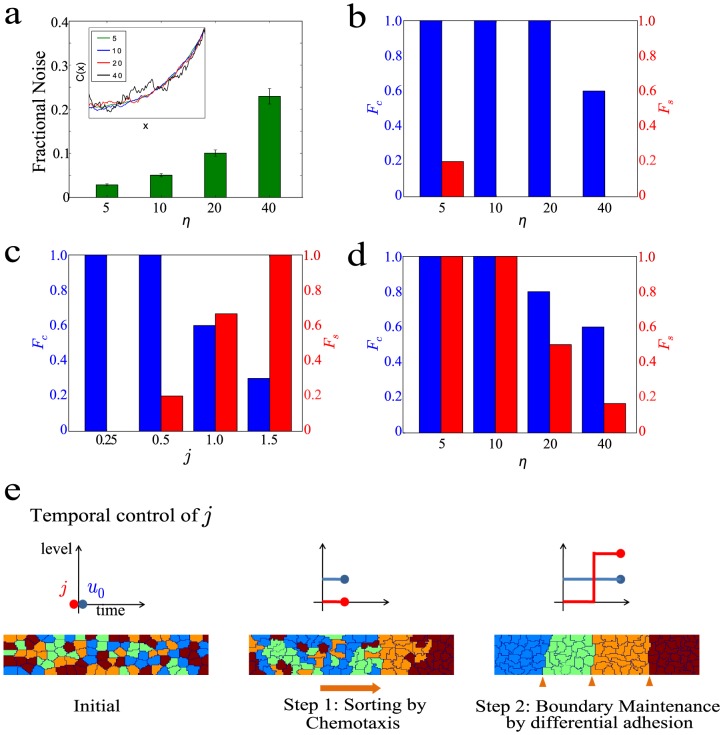
Chemotaxis coupled with delayed differential adhesion response yields optimal sorting in presence of noise. (**a**) Fractional noise in the morphogen gradient for different values of 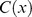. (Inset) Representative traces of morphogen concentration, 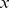, along 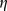 for different values of 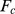. (**b**) Bar graphs of fraction of correct and stable sorting, 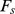 (blue) and 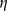
(red), respectively, for different values of 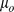 at 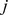 =  0.75 and 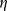  =  0.25. 10 runs were performed for each value of 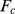. (**c**) Bar graphs of fraction of correct and stable sorting, 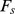 (blue) and 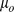 (red), respectively, for different values of *j*at 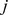 =  0.25 and *η*  =  10. 10 runs were performed for each value of 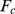. (**d**) Bar graphs of fraction of correct and stable sorting, 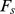 (blue) and 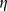
(red) respectively, for different values of 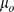 at 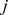 =  0.75 and 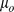  =  0 for the first 500 MCS, followed by 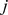 =  0.75 and 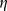  =  1.5 for the subsequent 2000 MCS. 10 runs were performed for each value of 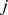. (**e**) Illustration for the chemotaxis coupled with delayed differential adhesion response model.

We found that intermediate values of 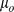 and 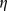 are optimal for achieving high fraction of correct and stable sorting in the absence of gradient noise. In this section, we explore the behavior of the system when noise was introduced into the morphogen gradient. We introduced different amount of fluctuations, characterized by the parameter 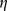, into the gradient. As shown in Fig. 5a (inset), the amount of variation in the gradient increases with 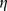. We quantified the absolute value of the fractional deviation of the gradient from the deterministic gradient for the various values of 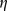 and found that we were in the range of fractional deviation between 0.025 to 0.2 (Fig. 5a). One of the few known careful measurements of morphogen noise, performed in *Drosophila* embryo for the Bicoid morphogen, yielded a fractional deviation of 0.1 which falls within the range of our study [36].

Using the optimal values of 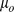 =  0.25 and 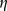 =  0.75 found in the previous section, we ran the stimulation for different values of 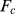. We determined the fraction of correct runs, 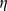, and found that it decreases with increasing 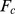 (Fig. 5b). This is expected as performance of cell sorting would deteriorate with increasing fluctuations in the gradient. We also found that although high 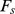 is achieved, the fraction of stable runs, 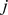 is low (Fig. 5b). This is likely due to spontaneous dissociation of correct clusters when exposed to gradient noise at the cell-type boundaries. Previously, we had shown that in the absence of gradient noise, differential adhesion response of 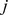 =  0.25 is able to prevent spontaneous dissociation from occurring (Fig. 4c). This suggests that higher values of 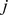 is needed to prevent spontaneous dissociation in the presence of noise.

We repeated the same analysis keeping *μ_o_* constant while increasing 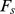. We found that although the fraction of stable runs, 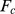, has increased, the fraction of correct runs, 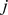 decreases (Fig. 5c). This result is similar to that discussed before whereby high differential adhesion leads to adhesion of cells of similar cell fate thus impeding correct migration of individual cells at the initial stages of cell sorting (Fig. 4a). In the absence of noise, there is a range of values for 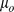 and 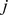 that is finely-tuned to satisfy the requirements for both correct sorting and stable cluster formation (Fig. 4c). In the presence of noise, it becomes more challenging to balance the forces of chemotaxis and differential adhesion to satisfy these requirements. Furthermore, values of 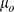 and 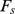 that yielded optimal sorting in the absence of noise do not do so now in the presence of noise. This suggests that a different strategy has to be adopted in the presence of noise.

Examining our computational model, we found that attaining correct and stable sorting requires two steps, migration and maintenance. In the migration step, cells sort out into their respective regions. In the maintenance step, cells have to "stick" tightly to like cells and not spontaneously dissociate from them. Viewing cell sorting as a two-step process allows us to think about the contributions of chemotaxis and differential adhesion at each step. In the first step, chemotaxis will be more important than differential adhesion as it acts as a systematic force to bias cells with different responses towards different ends of the tissue. Indeed if differential adhesion is too high, it may even impede the first step due to formation of stable clusters that do not sort correctly (Fig. 4d). In the second step, differential adhesion plays a more important role as it acts as a "glue" to keep cells of the same type together hence maintaining the clusters and preventing mixing. Hence we think a model with chemotaxis coupled with delayed differential adhesion response would lead to both high fraction of stable runs, 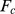, and high fraction of correct runs, 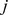.

To test the dynamical model, we stimulated cell sorting process for 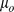 =  0 and 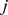 =  0.75 for 500 MCS, followed by 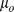 =  1.5 and 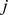 =  0.75 for the next 1500 MCS. We found that temporal control of 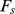 leads to high, 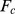 and 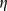 for a range of 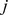 (Fig. 5d). This showed that temporal control of 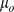 is an effective strategy leading to correct and stable cell sorting in the presence of noise.

## Discussion

We studied two mechanisms, chemotaxis and differential adhesion, to determine which led to better cell sorting. We first studied cell sorting in the absence of morphogen noise. We found that chemotaxis and differential adhesion conferred differing advantages to the sorting process; chemotaxis led to correct but unstable sorting, whereas differential adhesion resulted in low fraction of correct sorting which were very stable. The different outcomes of the chemotaxis and differential adhesion models led us to consider a model incorporating both mechanisms. We found that, in the absence of morphogen gradient noise, intermediate levels of the magnitude of chemotactic response, 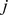, and the magnitude of differential adhesion, 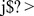, led to correct and stable sorting with low cell size variations. Next, we examined sorting in the presence of gradient noise and found that in the presence of morphogen gradient noise, chemotaxis coupled with delayed differential adhesion response can lead to optimal sorting.

There are other past computational work that studied the effects of combining chemotaxis and different adhesion [31], [32], [33]. Jiang *et al.* studied mound formation of *Dictyostelium discoideum* and suggested possible cooperation between differential adhesion and chemotaxis to bring about a tip containing only pre-stalk cells [31]. They found that differential adhesion aided in the sorting of pre-stalk cells to the surface of the mound whereas chemotaxis functioned in tip formation. Their finding that differential adhesion alone led to cell sorting differed from ours where both differential adhesion and chemotaxis were needed. This difference is likely due to the different geometries of mound formation versus sorting in a cell sheet. In mound formation, cells in the interior form more connections with other cells as they are completely surrounded by cells. On the other hand, cells on the surface of the mound form less cellular connections as they are also in contact with the surrounding. Since formation of more adhesive interactions are favored in order to minimize total energy of the system, the more adhesive cell type will form a cluster inside the less adhesive cell type. This leads to sorting of the less adhesive pre-stalk cells to the surface of the mould. This is different from sorting in a sheet where there is a-priori no difference between the left and right sides unless a chemotactic gradient is included. It would also be interesting to explore the time scale and stability of the sorting process in *Dictyostelium discoideum*.

Käfer *et al.* studied cell sorting in the presence of both differential adhesion and chemotaxis [32]. Their work aimed to provide a general framework for understanding cell rearrangement not specific for a particular biological context. Unlike our model, they considered homogeneous chemotactic response where both cell types responded similarly to the chemotactic gradient. Similarly, they also found that cell sorting occurs much faster with chemotaxis and observed a size-based segregation of the cells. Furthermore, they found that cells could move against the direction of chemotaxis. This was similar to our observation that even when a cell was attracted to the morphogen, it could move against the direction of chemotaxis as it was being pushed away by other types of cells experiencing greater attraction to the morphogen (Fig. S2 in File S1). Lastly, Käfer *et al.* concluded that during cell sorting, increasingly larger clusters of cells were formed and these clusters could change the local neighbourhood of individual cells thus affecting sorting outcome. This was similar to what we observed in Fig. 4d where big clusters that were formed during sorting impeded the sorting process and affected the final outcome. Our present work differs from this work as follows. In Kafer et al., the cells were always randomly distributed initially whereas in our model with differential adhesion alone, we started with an initial system of only a few misspecified cells. We initialized our system this way as our differential adhesion model is one which allowed for cell sorting followed by imperfect cell fate specification to a noisy morphogen gradient. Hence, we were able to show that even if most of the cells were patterned correctly initially, differential adhesion is still not robust enough to drive the few misspecified cells out to the correct region. Also, because of our specific biological context of tissue patterning in mind, we can quantify metrics like fraction of correct sorting, fraction of stable sorting and sorting time very carefully, whereas these metrics are either not determined nor emphasized in Kafer *et al.* We placed a lot of emphasis on these quantities relating to stability of the clusters and time spent in sorting because robustness of boundary maintenance and developmental time frame are pertinent issues when studying pattern formation.

Finally, Zhang *et al.* studied in detail how the choice of cell adhesion molecule binding affects cell sorting [33]. Their study helped connect signaling models at the molecular level to cell sorting and tissue patterning at the tissue-level.

Unlike our results in the absence of morphogen gradient, a complicated dynamical interplay of chemotaxis and delayed differential adhesion is required for cell sorting in the presence of noise. We propose that cells first express molecules involved in chemotaxis, followed by surface molecules involved in differential adhesion for effective control for cell sorting. Dynamic gene expression has been observed in development and we foresee that future time-lap microscopy experiments following the expression of molecular players will yield greater insight into the sorting process. Future work is also needed to identify the molecular players involved in execution of these differential adhesion and chemotactic response. Differential adhesion can be mediated by having the cell types expressing different or different combinations of surface molecules. Possible candidates include cadherins and photocadherins which have been found to be expressed in conserved domains along the dorsoventral axis in the spinal cord of chicken embryo [37].

In *Dictyostelium* and mammalian cells, activation of PI3 kinase (PI3K) and the downstream Akt have been found to be responsible for the coordinated regulation of the actin cytoskeleton leading to chemotaxis [38]. It will be interesting to determine if these pathways are also involved in the chemotactic response and they can be activated differently to generate different chemotactic responses in the different cell types. The identity of the the morphogen for chemotaxis is currently unclear. In the zebrafish neutral tube, Xiong *et al.* found that Sonic hedgehog is not required for sorting, suggesting that noncanonical Sonic hedgehog or other molecules like Wnt may be responsible for cell sorting [21].

Currently, experiments to identify proteins functional in sorting, usually involved single protein knockout. Our work suggests that knockout of two proteins, one involved in differential adhesion and another in chemotaxis, may be required to observe a more severe phenotype.

Here, we studied mechanisms for cell sorting to yield sharply delineated domains of cell types. Recently, works on striped pattern formation in zebra fish and hair placode morphogenesis also suggested the importance of cell migration in formation of these other kinds of patterns [39], [40]. These experiments show that there is an increasing need to view patterning as a dynamical process in space. Future work incorporating signaling with cell sorting models will be useful in understanding other patterning processes.

## Supporting Information

File S1 **Supplementary figures and tables.**
(PDF)
