# Supplementary material for: Computational Modeling Reveals that a Combination of Chemotaxis and Differential Adhesion Leads to Robust Cell Sorting during Tissue Patterning
Source: PLoS One. 2014 Oct 10;9(10):e109286. doi: 10.1371/journal.pone.0109286 (PMC4193783; doi:10.1371/journal.pone.0109286)
Supplement: File S1 — Supplementary figures and tables. (PDF) [file pone.0109286.s001.pdf]

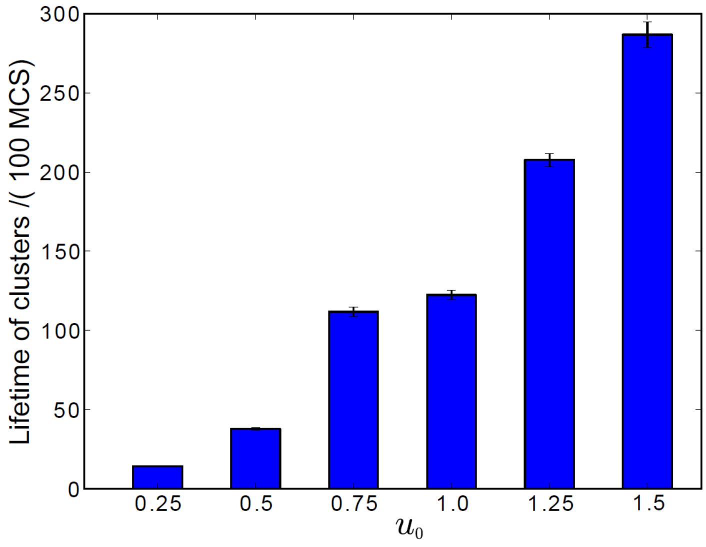

Lifetime of clusters /(100 MCS)
300
250
200
150
100
50
0
0.25
0.5
0.75
1.0
1.25
1.5
u_0

Fig. S1

Attractive: $\mu_\tau = \mu_o \begin{pmatrix} 0.25 \\ 0.5 \\ 1 \\ 2 \end{pmatrix}$

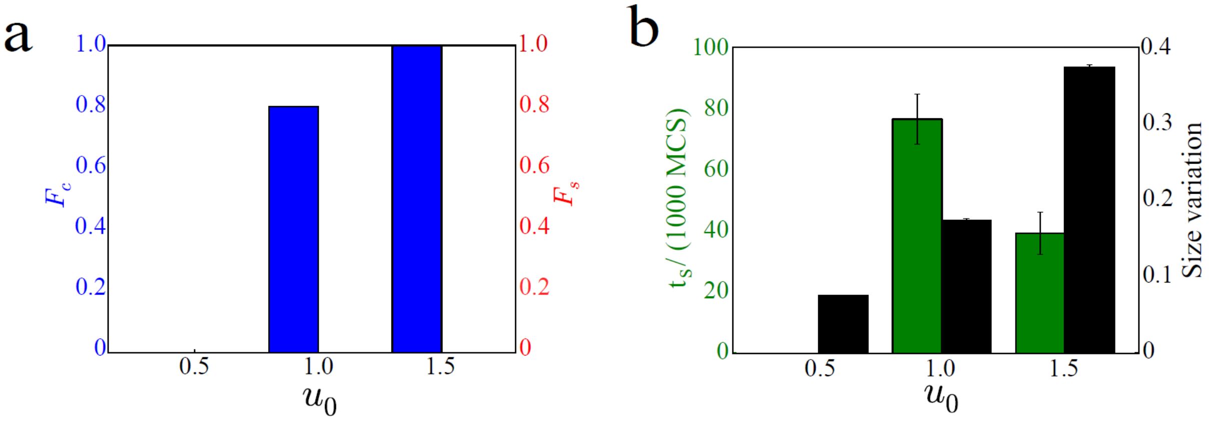

Repulsive: $\mu_\tau = \mu_o \begin{pmatrix} -4 \\ -2 \\ -1 \\ -0.5 \end{pmatrix}$

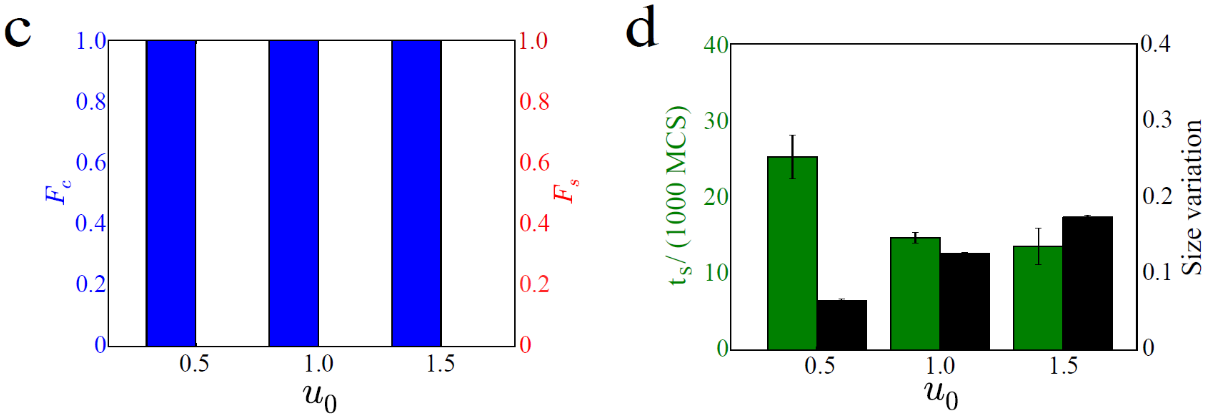

Fig. S2

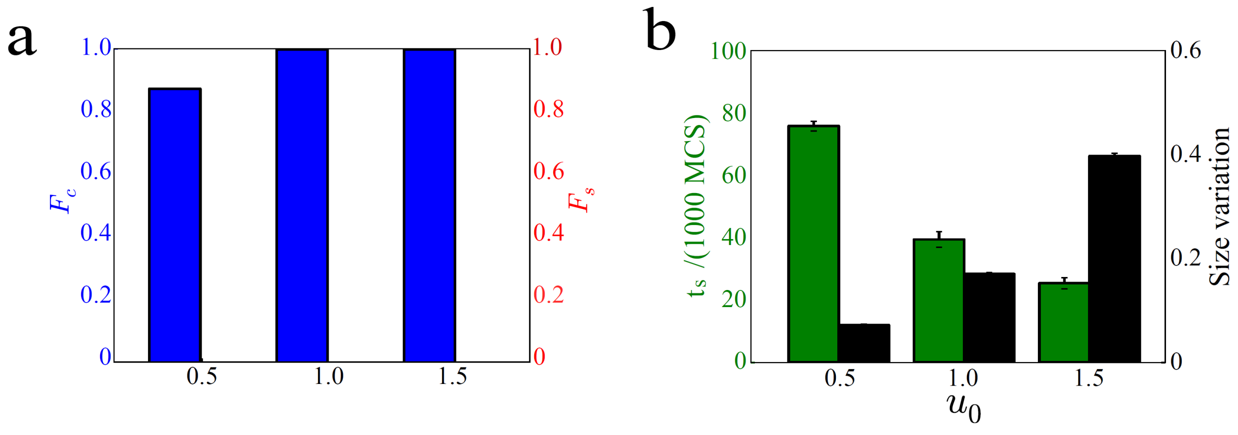
a
1.0
0.8
0.6
0.4
0.2
0
F_c
F_s
0.5
1.0
1.5
b
100
80
60
40
20
0
t_s /(1000 MCS)
0.6
0.4
0.2
Size variation
u_0

Fig. S3

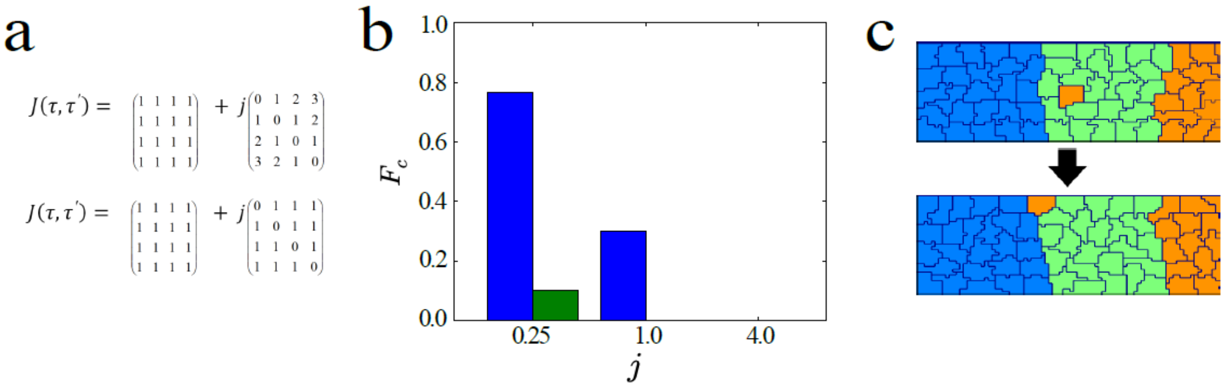
a
$J(\tau,\tau') = \begin{pmatrix}1&1&1&1\\1&1&1&1\\1&1&1&1\\1&1&1&1\end{pmatrix} + j\begin{pmatrix}0&1&2&3\\1&0&1&2\\2&1&0&1\\3&2&1&0\end{pmatrix}$
$J(\tau,\tau') = \begin{pmatrix}1&1&1&1\\1&1&1&1\\1&1&1&1\\1&1&1&1\end{pmatrix} + j\begin{pmatrix}0&1&1&1\\1&0&1&1\\1&1&0&1\\1&1&1&0\end{pmatrix}$
b
1.0
0.8
0.6
0.4
0.2
0.0
F_c
0.25
1.0
4.0
j
c

Fig. S4

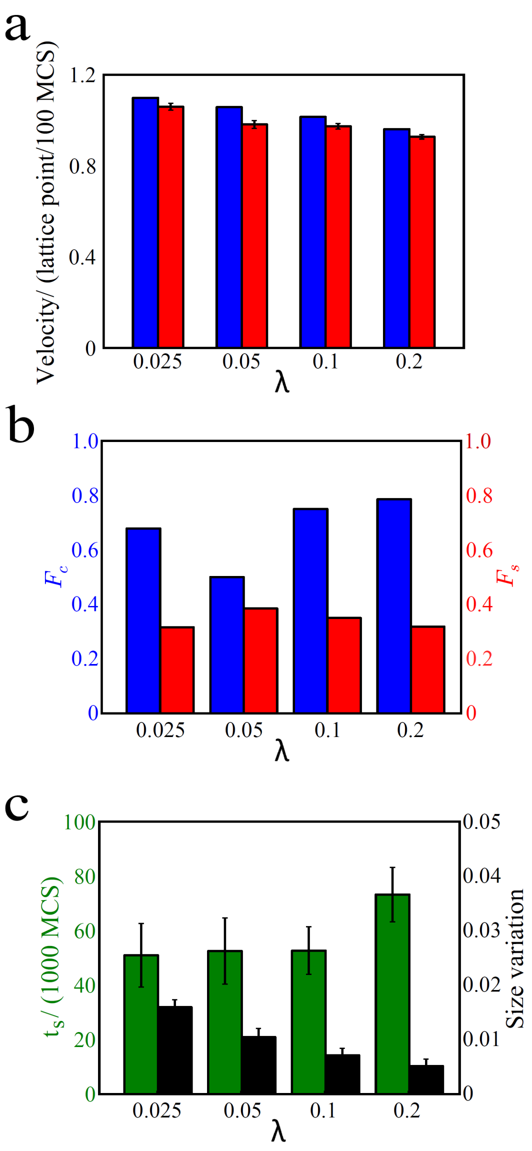

a
Velocity/ (lattice point/100 MCS)
1.2
0.8
0.4
0
0.025
0.05
0.1
0.2
λ
b
1.0
0.8
0.6
0.4
0.2
0
F_c
F_s
0.025
0.05
0.1
0.2
λ
c
100
80
60
40
20
0
t_S/ (1000 MCS)
0.05
0.04
0.03
0.02
0.01
0
Size variation
0.025
0.05
0.1
0.2
λ

Fig. S5

j \ μ_o	0	0.25	0.5	0.75	1	1.25	1.5
0	-	-	-	-	-	-	-
0.25	-	0.52	-	-	-	-	-
0.5	-	0.70	0.38	-	-	-	-
0.75	-	0.73	0.52	0.87	0.36	0.97	0.11
1	-	-	0.54	0.44	0.89	0.63	0.12
1.25	-	-	0.74	0.67	1.00	0.13	0.53
1.5	-	-	0.70	0.38	-	0.44	0.89

(Table S1a)

j \ μ_o	0	0.25	0.5	0.75	1	1.25	1.5
0	-	0.55	0.92	0.06	0.01	0.02	0.57
0.25	-	0.53	0.83	0.56	0.58	0.57	0.92
0.5	-	0.40	0.98	0.32	0.91	0.32	0.61
0.75	-	-	0.29	0.67	0.27	0.56	0.38
1	-	-	0.62	0.58	0.42	0.96	0.91
1.25	-	-	0.37	0.78	0.39	0.54	0.21
1.5	-	-	-	0.62	-	0.40	0.79

(Table S1b)

SUPPORTING INFORMATION LEGENDS:

Fig. S1: Lifetime of clusters for chemotaxis model. Bar graphs of the lifetime of clusters for different values of μ_o.

Fig. S2: Chemotaxis model with pure attractive or repulsive response can lead to high fraction of correct sorting. (a) Bar graphs of fraction of correct and stable sorting, F_c (blue) and F_s (red), respectively, for different values of μ_o for attractive response. None of the runs led to stable sorting. 5 runs were performed for each value of μ_o **(b)** Bar graphs for sorting time, t_s, (green) and size variation (black) for different values of μ_o for attractive response. Error bars show the standard errors. **(c)** Bar graphs of fraction of correct and stable sorting, F_c (blue) and F_s (red), respectively, for different values of μ_o for repulsive response. None of the runs led to stable sorting. 5 runs were performed for each value of μ_o **(d)** Bar graphs for sorting time, t_s, (green) and size variation (black) for different values of μ_o for repulsive response. Error bars show the standard errors.

Fig. S3: Chemotaxis model for higher number of cells. (a) Bar graphs of fraction of correct and stable sorting, F_c (blue) and F_s (red), respectively, for different values of μ_o for attractive response. None of the runs led to stable sorting. 8 runs were performed for each value of μ_o **(b)** Bar graphs for sorting time, t_s, (green) and size variation (black) for different values of μ_o for attractive response. Error bars show the standard errors.

Fig. S4: Differential adhesion model with similar differential adhesion towards unlike cells lead to lower fraction of correct sorting. (a) Surface energy per unit contact area relationship among the four types of cells for two different models. Top: model with differentiation towards unlike cells. Bottom: model with no differentiation towards unlike cells. **(b)** Bar graphs of fraction of correct sorting, F_c, for different values of magnitude of differential adhesion, j, for model with (blue) and without (green) differentiation. 30 runs were performed for model with differentiation and 10 runs were performed for model without differentiation for each value of j. **(c)** Cell grid obtained for model without differentiation.

Fig. S5: Varying λ does not affect the fraction of correct and stable sorting, F_c and F_s. (a) Bar graphs showing the speeds of correctly specified (blue) and incorrectly specified cells (red) for different values of λ. **(b)** Bar graphs of fraction of correct and stable sorting, F_c (blue) and F_s (red), respectively, for different values of λ. **(c)** Bar graphs for sorting time, t_s, (green) and size variation (black) for different values of λ. Error bars show the standard errors.

Table S1: Initial number of clusters does not affect sorting outcome and sorting time. (a) P-values obtained when applying a 2 sample t-test to determine if the initial number of clusters for runs that lead to correct and incorrect sorting are the same. The t-test is performed for different values of j and μ_o, The null hypothesis cannot be rejected for all the values of j and μ_o tested. **(b)** P-values obtained when applying a spearman test to determine correlation between the initial number of clusters and sorting time for runs that sort correctly. The spearman test is performed for different values of j and μ_o, The null hypothesis cannot be rejected for all the values of j and μ_o tested.
